# A4 *SACCHAROMYCES BOULARDII* CNCM I-745 ENHANCES DUODENAL MICROBIOTA-MEDIATED TRYPTOPHAN METABOLISM IMPROVING GLUTEN IMMUNOPATHOLOGY

**DOI:** 10.1093/jcag/gwae059.004

**Published:** 2025-02-10

**Authors:** K Kan, M Constante, S Rahmani, A Caminero, X Roux, H Galipeau, E Verdu

**Affiliations:** McMaster University, Hamilton, ON, Canada; McMaster University, Hamilton, ON, Canada; McMaster University, Hamilton, ON, Canada; McMaster University, Hamilton, ON, Canada; Biocodex Centre de recherche, Compiegne, France; McMaster University, Hamilton, ON, Canada; McMaster University, Hamilton, ON, Canada

## Abstract

**Background:**

Impaired microbial indole production and decreased activation of the aryl hydrocarbon receptor (AhR) pathway has been reported in active coeliac disease (CeD). However, *whether* and *how* this can be targeted by specific microbial therapies is unknown.

**Aims:**

We tested whether *Saccharomyces boulardii* CNCM I-745 (*Sbou*) influences indole production and AhR activation through stimulation of microbial enzymatic activity.

**Methods:**

Transgenic NOD/DQ8 mice were immunized with partially digested gliadin and cholera toxin weekly for 3 weeks followed by gluten challenges (10 mg; alternate days/3 weeks). Non-sensitized mice were used as controls. Mice were treated daily with 3 g/kg of *Sbou* or water during the study. Additional immunized mice treated or not with *Sbou*, received daily gavage with AhR antagonist CH-223191 (10 mg/kg) or vehicle. Small intestinal pathology was analysed by villus to crypt (V/C) ratios and CD3^+^ intraepithelial lymphocyte (IEL) counts. Expression levels of the AhR-activated gene Cyp1a1 were determined by RT-qPCR. Small intestinal microbiota of NOD/DQ8 mice and CeD patients were cultured in BHI media supplemented with 10 g/L Gelatin or Casein as additional protein sources with and without 1x10^6^*Sbou* CFUs. Free tryptophan and AhR activation were assayed using a commercial kit and a transfected reporter cell line, respectively. Resulting microbiota was assessed by 16S rRNA gene sequencing and PICRUSt-predicted functions.

**Results:**

*Sbou* increased *Cyp1a1* levels, mucosal tryptophan and AhR activation in gluten immunized mice. This was paralleled by improved V/C ratios and lower IELs *vs* no probiotic, which was inhibited by AhR antagonist. In immunized mice treated with *Sbou* microbiota analysis revealed higher expression of genes involved in protein digestion and lower tryptophan 2,3-dioxygenase, a tryptophan degrader. Mouse duodenal microbiota cultures treated with *Sbou* had higher proteolytic activity but lower tryptophan 2,3-dioxygenase, paralleled by higher tryptophan *vs* cultures with no probiotic. In duodenal microbiota cultures from CeD patients *Sbou* increased tryptophan *vs* no probiotic.

**Conclusions:**

*S. boulardii* CNCM I-745 reduced gluten immunopathology in immunized NOD/DQ8 mice through activation of the AhR pathway. *In vitro* experiments suggest *Sbou* synergises with the microbiota to increase free tryptophan from protein sources and inhibit tryptophan degradation, leading to higher indole production and AhR activation. The results identify a specific mechanistic pathway by *S. boulardii* CNCM I-745 in celiac disease and should encourage clinical testing in this population.

**Funding Agencies:**

CIHRBiocodex, France

